# Demyelinating Disease of the Central Nervous System Concurrent With COVID-19

**DOI:** 10.7759/cureus.17297

**Published:** 2021-08-19

**Authors:** Sibel Karsidag, Sevki Sahin, Miruna F Ates, Nilgun Cinar, Sude Kendirli

**Affiliations:** 1 Neurology, Maltepe University, School of Medicine, Istanbul, TUR

**Keywords:** covid-19, demyelinating disease, multiple sclerosis, adem, demiyelination

## Abstract

Neurological diseases related to coronavirus disease-2019 (COVID-19) are increasingly reported. We report here three cases that presented with subtle neurologic findings manifesting within a range of 15 days to four months after their COVID-19 diagnoses. Magnetic resonance imaging showed acute multifocal periventricular and subcortical demyelinating lesions. Some of the lesions showed contrast enhancement and diffusion restriction. Severe acute respiratory syndrome coronavirus 2 (SARS-CoV-2) PCR was found in the cerebrospinal fluid of just one patient. All patients received intravenous methylprednisolone therapy. In this report, we aim to discuss the aspects of possible COVID-19-related demyelination that support a diagnosis of multiple sclerosis (MS) or acute disseminated encephalomyelitis (ADEM).

## Introduction

Novel coronavirus 2019 is a neurotropic virus that can cause complications in the central (CNS) and peripheral nervous system (PNS). Neurological involvement can be seen in 36% of patients with COVID-19. These complications include headache, vertigo, acute cerebrovascular disease, encephalopathy, ataxia, demyelinating disease, and polyneuropathies [1]. Involvement patterns of the central nervous system (CNS) related to COVID-19 such as acute disseminated encephalomyelitis (ADEM), ADEM-like syndrome, ischemic or vasculitic stroke, and multiple sclerosis-like disorders have been reported [2].

Herein, we aimed to demonstrate all aspects of three cases of demyelinating disease that developed in the post-COVID-19 period.

## Case presentation

Case 1

**Figure 1:**
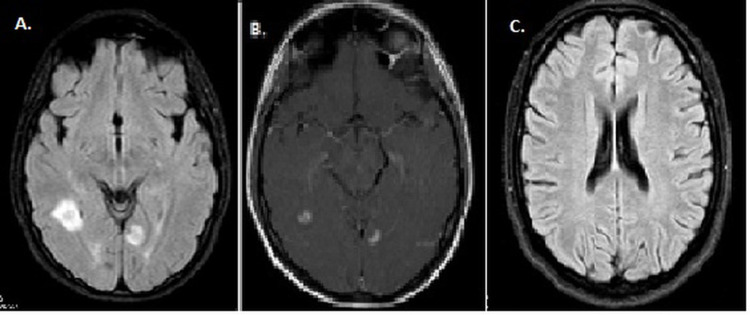
Case 1 Brain MRI revealed hyperintense periventricular and juxtacortical lesions in an axial FLAIR sequence (A) and contrast enhancements in axial T1 sequence (B). After two months, it was observed that the lesions regressed in the FLAIR sequence (C).

A previously healthy 18-year-old female was admitted to the neurology outpatient clinic for dizziness and imbalance. She had fever, diarrhea, fatigue, and hyposmia over the past 15 days. Her polymerase chain reaction (PCR) for SARS-CoV-2 was positive from the nasopharyngeal swap and she received favipiravir 3200 mg on Day 1, followed by 600 mg twice a day from Days 2-5. Neurological examination showed horizontal nystagmus, truncal ataxia, and cerebellar dysmetria on both sides. A magnetic resonance imaging (MRI) showed multiple hyperintense ovoid and round lesions in T2-weighted and fluid-attenuated inversion recovery (FLAIR) sequences in periventricular and subcortical white matter. Lesions of abnormal high signal intensity on diffusion-weighted images (DWI) and apparent diffusion coefficient (ADC) maps were seen. Some lesions had contrast enhancement with gadolinium (Figure 1). Cerebrospinal fluid (CSF) analysis demonstrated no cells, protein 11 mg/dl (normal 15 - 45 mg/dl), glucose 52 mg/dl (normal 40 - 70 mg/dl). Bacterial, fungal culture, viral panel for herpes simplex virus (HSV), Epstein-Barr virus (EBV), and cytomegalovirus (CMV) were found negative. Oligoclonal bands (OCBs) were found negative and the IgG index was 1.22. CSF PCR for SARS-CoV-2 was negative. After her ataxia improved, she started to walk independently after high dose intravenous corticosteroid treatment (methylprednisolone 1g/day for seven days). A follow-up MRI was completely normal after two months.

Case 2

**Figure 2:**
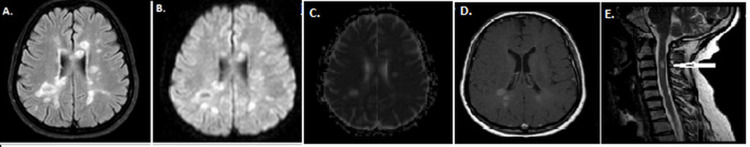
Case 2 MRI showed multiple, periventricular, bilateral, symmetrical, ovoid and round, prone to confluent, hyperintense lesions in an axial FLAIR (A) restriction on DWI (B), the trace apparent diffusion coefficient (ADC) in some lesions (C), and contrast enhancement in axial T1 sequence (D).  A contrast-enhancing hyperintense lesion covering one segment was observed on a sagittal T1 image of cervical spine MRI (E).

A 42-year-old female received favipiravir treatment with the diagnosis of COVID-19 one month ago. She was admitted to the neurology outpatient clinic with jaw and left facial pain for 10 days. Her neurological examination showed paresthesia in the left mandibular branch of the trigeminal nerve, 4/5 paresis in left ankle dorsiflexion, and the Babinski sign was found positive on the left foot. Brain MRI showed multiple, periventricular, bilateral, ovoid, punctate, prone to confluent, hyperintense lesions on the T2 and FLAIR images. These lesions had diffusion restriction on DWI with increased ADC map. Some lesions showed contrast enhancement. At the same time, a contrast-enhancing hyperintense lesion covering 1 segment was observed on cervical MRI (Figure 2). Serum biochemical analyses were normal except for a minimally elevated sedimentation rate (30 mm/h) and C-reactive protein (CRP) (5.3 mg/). CSF examination revealed no cells, protein 22 mg/dl (normal 15 - 45 mg/dl), glucose 48 mg/dl (normal 40 - 70 mg/dl), OCBs negative, and an IgG index of 0.9. Antinuclear antibody (ANA), extractable nuclear antigen antibodies (ENA), Brucella-IgG, IgM antibody, Mycobacterium tuberculosis PCR, syphilis IgG/IgM, human immunodeficiency virus (HIV), aquaporin-4 (AQP4)-IgG were found negative. CSF PCR for SARS-CoV-2 was negative. Bacterial and fungal culture and the viral PCR panel for HSV, EBV, CMV were found negative. Transthoracic echocardiogram was normal. Methylprednisolone 1g/day IV was administered for seven days. Paresthesias in the mandibular branch of the trigeminal nerve and paresis in the left ankle dorsiflexion improved.

**Figure 3:**
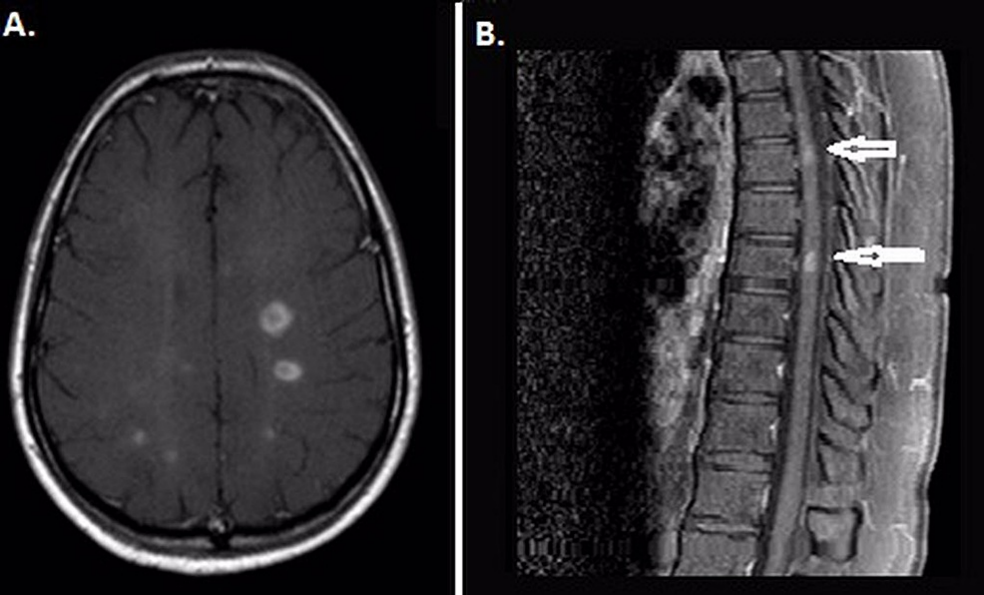
Case 2 five months later MRI After five months, note the development of new contrast-enhancing lesions in the centrum semiovale (A) and thoracal spinal cord (B).

Five months after the first attack, there was mild weakness in both legs, sensory disturbance, and paresthesia at the T8 nerve root level and below with bladder incontinence. The MRI demonstrated new abnormal signal intensities with contrast enhancement in both thoracal 6 and 8 levels and brain (Figure 3). CSF and nasal swab PCR for SARS-CoV-2 were found negative. Methylprednisolone at 1g/day IV for 10 days was administered. Weakness in both legs, hypoesthesia level below T8, and bladder incontinence were improved.

Case 3

**Figure 4:**
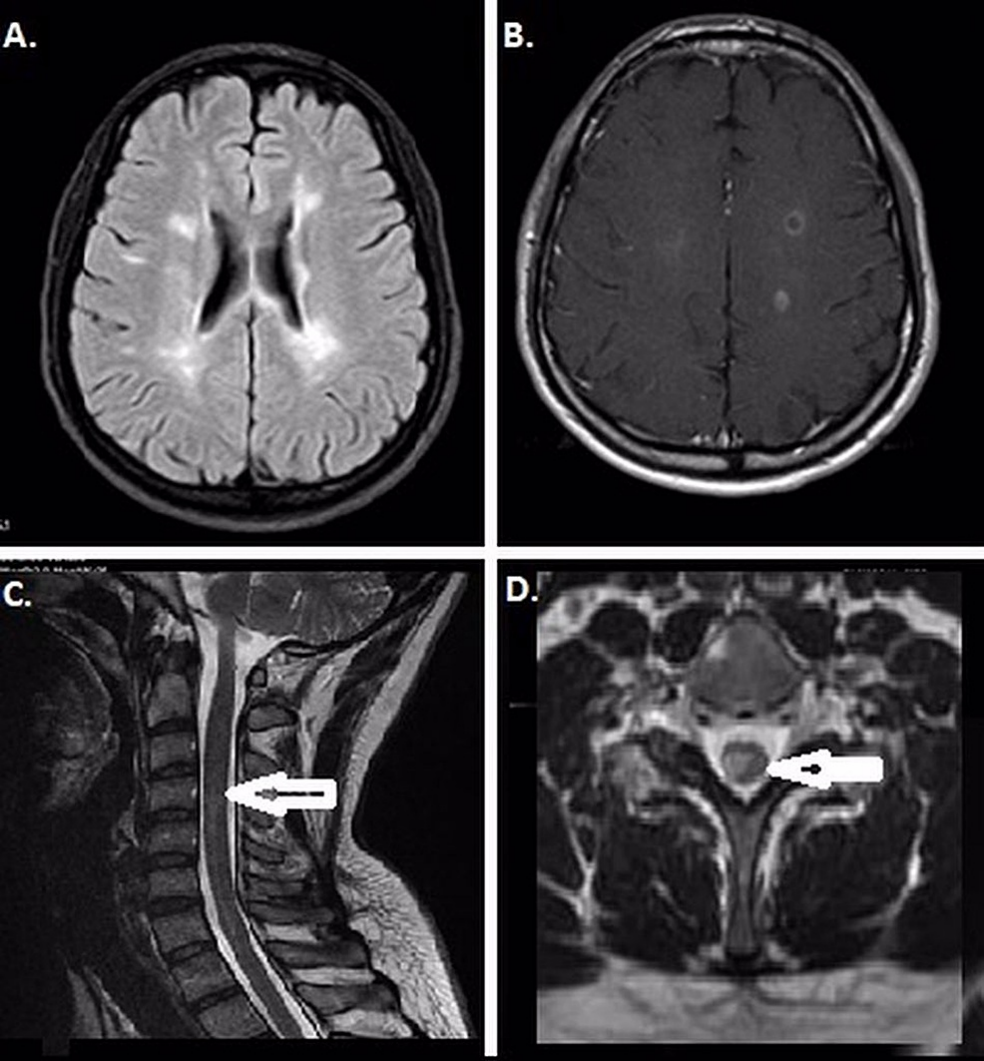
Case 3 Hyperintense lesions in the periventricular area in an axial FLAIR (A), and contrast enhancement lesions involving centrum semiovale in axial T1 sequences (B). After two months, note the new contrast-enhancing lesions in the cervical spinal cord in sagittal (C) and axial T2 sequences (D).

A previously healthy 32-year-old male was admitted to the neurology outpatient clinic with numbness in the left half of his jaw for one week. The patient stated that he had a COVID-19 infection four months previously and underwent a favipiravir 8.000 mg treatment over five days. A neurological examination showed hypoesthesia in the mandibular branch of the trigeminal nerve. Routine biochemical examinations were normal. Periventricular, ovoid hyperintensities, and punctate lesions in the FLAIR and T2 sequences on MRI were seen. Some of them showed contrast enhancement (Figure 4A-4B). There were also lesions in the cerebellum and left pontocerebellar junction. Some lesions had diffusion restriction on DWI with increased trace ADC map. The patient did not accept hospitalization, therefore an initial course of 64 mg of oral methylprednisolone was started and slowly tapered off over three weeks. Neurological symptoms were completely resolved. After two months, he developed weakness in the right leg. Neurologic examination showed 4/5 weakness, hyperexcitable deep tendon reflexes, and Babinski sign on the right side. An MRI demonstrated new abnormal signal intensities with contrast enhancement at the C 6-7 levels. (Figure 4C-4D). CSF revealed no blood cells, protein 30 mg/dl (normal 15 - 45 mg/dl), glucose level 80 mg/dl (normal 40 - 70 mg/dl), positive oligoclonal band (Type II) and the IgG index was 1.01. Antinuclear antibody (ANA), extractable nuclear antigen antibodies (ENA), Brucella-IgG antibody, Mycobacterium tuberculosis PCR, Syphilis IgG/IgM, HIV were found negative. CSF PCR for SARS-CoV-2 was positive. Bacterial, fungal, and viral PCR panels for HSV, EBV, CMV were found negative. Methylprednisolone 1g/day IV for 10 days was administered and weakness in both legs was improved.

## Discussion

The coronavirus family has a high neurotropism and COVID-19 is a potent virus for the nervous system [1]. The direct invasion of the virus into the brain from the nasopharyngeal area, the disruption of the blood-brain barrier integrity by cytokines, and the affinity of the virus for angiotensin-converting enzyme 2 (ACE2) receptors can cause nervous system involvement [3]. COVID-19 can induce an inflammatory response with glial cell activation and demyelination by causing a pro-inflammatory process. Demyelinating diseases might develop in the early or late stage of COVID-19 in the CNS [3].

Palao et al. presented a patient affected by COVID-19 and who developed decreased visual acuity. The MRI of this patient showed inflammation in the optic nerve and demyelinating lesions in the CNS. Because non-enhancing and enhancing periventricular lesions were found together, researchers assumed that COVID-19 was a precipitating factor for multiple sclerosis in this patient [4].

Parsons et al. reported a case of ADEM due to COVID-19. The MRI of this patient demonstrated acute multifocal demyelinating lesions. They described that CSF PCR for SARS-CoV-2 was negative. Although clinic findings of this patient improved, follow-up MRI showed that lesions had increased in number and distribution [5].

Zanin et al. described a COVID-19 patient admitted for interstitial pneumonia and seizures. It is reported that this patient's status had deteriorated and intubation was needed. They reported that a brain MRI of this patient showed multiple hyperintense lesions in T2WI, without the restriction of diffusion nor contrast enhancement in the periventricular white matter, bulbo-medullary junction, cervical and dorsal spinal cord. They stated that delayed immune response after the viremia can cause demyelinating lesions in the CNS [6].

ADEM cases related to COVID-19 were reported associated with serious clinical findings [6,7]. Autopsy studies showed the presence of demyelination and SARS-CoV-2 virus particles in the brain of COVID-19 patients [8].

In the cases illustrated above, the CSF SARS-CoV-2 PCR was found negative. In our current report, only one case showed PCR positivity in CSF. Subsequent reactivation of SARS-CoV-2 or reinfection with other SARS-CoV-2 strains is a problem. Prolonged median SARS-CoV-2 RNA shedding time was reported as 53 days, a maximum of 83 days in 36 patients [9]. Some authors reported that recurrence was related to prolonged carriage in patients recovering from COVID-19. A later positive PCR rate was reported as 14.5%-21% in negative patients [10]. In asymptomatics, it has been suggested that the fecal-oral route can be a possible route of transmission. SARS-CoV-2 PCR was not detected on a nasopharyngeal swab but found positive on an anal swab on the 42nd day [11].

In the third recovered (four months previously) COVID-19 patient, positive SARS-CoV-2 PCR was found in the CSF despite it being negative in the nasopharyngeal swab. The presence of concurrent acute brain and medullary lesions may be a warning of prolonged SARS CoV-2 RNA shedding time (120 days) and a reactivation of the virus in body fluids.

Multifocal hyperintensities in deep white matter and juxta cortical areas in MRI are seen in ADEM. The periventricular regions are usually spared. Lesions are commonly symmetrical and patchy appearance [12]. Some of the lesions may show diffusion restriction. In adults, ADEM can be difficult to differentiate from the first attack of MS, based only on radiographic findings [13]. Clinical findings in ADEM contain a history of prodromal viral illness, fever, nuchal rigidity, ataxia, and impaired consciousness, and/or encephalopathy. ADEM rarely presents with subtle findings although it has generally severe multifocal neurologic findings [13]. It is classically a monophasic disorder. Symptoms begin within two days to four weeks after antigenic stimulation. New lesions do not develop in monophasic ADEM. In recent years, forms of recurrent demyelination called multiphasic ADEM have also been described [13].

Multiple sclerosis is characterized by a relapsing-remitting course and periventricular and juxtacortical lesions spreading over both time and space [14]. However, there can be an overlap between ADEM and MS regarding CNS symptoms and radiographic evidence. The ratio of patients diagnosed with MS in further follow-up, despite the first diagnoses being ADEM, is reported as 9.5-27% [15].

In our patients, the presence of a single attack in the first case, two attacks in the second and third cases, the presence of plaques that are distributed in time and space in MRI (contrast-enhancing and non-contrast lesions), and the high IgG index support a diagnosis of MS according to McDonald criteria [14].

In our cases, demyelinating lesions developed after the viral infection from another point of view. The diagnosis of the first patient may be ADEM but the possibility of the first presentation of MS should also be kept in mind. In the other cases, the presence of recurrence suggests the possibility of MS. Also, an increased frequency of relapses due to CoVID-19 in MS patients is rarely reported [16].

However, the plaques on our patients’ MRIs have atypical appearances. Some of them are larger than typical MS plaques and include the deep structures as well as the periventricular and juxtacortical regions. Yavari et al. also pointed out that demyelinating lesions due to COVID-19 are different from MS plaques [17].

A further finding in the MRIs of our patients is the presence of small punctate lesions. The presence of both punctate lesions and demyelinating lesions have been thought to be related to small vessel vasculitis caused by COVID-19. Varga et al. showed that diffuse endothelial inflammation developed with the invasion of the virus, the blood-brain barrier was disrupted, and inflammation and cerebral ischemia coexisted [18].

All of our patients have diffusion restrictions in some of their MRI lesions. It is reported that diffusion coefficients of acute MS plaques are increased. The elevation in DWI and ADC is related to vasogenic edema [19]. In all of our patients, it was found that the lesions showed diffusion restriction on DWI with an increased ADC map. This is to support vasogenic edema in those lesions.

Peripheral hyperintense open and closed ring contrast enhancement was seen in our patient MRIs. Such lesion appearances in MRIs usually point to a demyelinating lesion [20]. In MS, the classical inflammatory cascade develops on the venule’s side of the microcirculation and spreads in the parenchyma surrounding the small parenchymal venules [21]. Conversely, inflammatory vasculopathy affects medium and small vessels, usually the arteriolar side of cerebral microcirculation [12]. By linking the clinic and MRI findings, it seems logical to conclude that COVID-19 infection may result in demyelination, inflammation, and small-vessel vasculitis affecting myelin sheaths.

The remarkable findings in our patients were as follows: Although many multiple lesions were detected, the clinical presentations of cases were mild. This condition can sometimes be reported in MS. Another finding is a short time interval between COVID-19 infection and cerebral lesions. This finding might support the diagnosis of ADEM. Their follow-up will determine whether the diagnosis in our patients is MS or ADEM.

These cases with subtle clinical findings could be regarded as ADEM triggered by COVID-19 or could represent a potential transformation into MS due to relapses.

## Conclusions

Subtle neurological findings should not be ignored in patients with COVID-19, and imaging studies should be repeated. Reinfection with other SARS-CoV-2 strains, reactivation of the virus in body fluids, and prolonged median SARS-CoV-2 RNA shedding time should be kept in mind. Recurrent demyelinating lesions in patients with COVID-19 require follow-up as a signal of conversion to MS. Long-term and observational studies are required.
